# Supplementary material for: The association between material hardship and physical and mental health among older adults: Multi-channel sequence Approach
Source: PLoS One. 2025 Mar 10;20(3):e0319270. doi: 10.1371/journal.pone.0319270 (PMC11893115; doi:10.1371/journal.pone.0319270)
Supplement: S1 Table — (DOCX) [file pone.0319270.s001.docx]

**S1 Table. Regression analysis results with depressive symptoms**

| Reference group | | 1. Least materially burdened | 2. Multiply burdened | 3. Financially burdened | 4. Housing cost-burdened | 5. Financial & housing cost-burdened |
| --- | --- | --- | --- | --- | --- | --- |
|  | 1. Least materially burdened | - | 0.68***  (0.45 - 0.9) | 0.28**  (0.09 - 0.47) | 0.14  (-0.04 - 0.35) | 0.44***  (0.18 - 0.7) |
|  | 2. Multiply burdened | -0.68***  (-0.9 - -0.45) | - | -0.4**  (-0.64 - -0.15) | -0.53***  (-0.78 - -0.29) | -0.23  (-0.53 - 0.07) |
|  | 3. Financially burdened | -0.28**  (-0.47 - -0.09) | 0.4**  (0.15 - 0.64) | - | -0.13  (-0.35 - 0.08) | 0.16  (-0.12 - 0.45) |
|  | 4. Housing cost-burdened | -0.14  (-0.33 - 0.04) | 0.53***  (0.29 - 0.78) | 0.13  (-0.08 - 0.35) | - | 0.3*  (0.02 - 0.58) |
|  | 5. Financial & housing cost-burdened | -0.44***  (-0.7 - -0.18) | 0.23  (-0.07 - 0.53) | -0.17  (-0.45 - 0.12) | -0.3*  (-0.58 - -0.02) | - |

*** p ≤0.001; ** p ≤0.01; * p ≤ 0.05
