# Supplementary material for: The association between material hardship and physical and mental health among older adults: Multi-channel sequence Approach
Source: PLoS One. 2025 Mar 10;20(3):e0319270. doi: 10.1371/journal.pone.0319270 (PMC11893115; doi:10.1371/journal.pone.0319270)
Supplement: S2 Table — (DOCX) [file pone.0319270.s002.docx]

**S2 Table. Regression analysis results with self-rated health**

| Reference group | | 1. Least materially burdened | 2. Multiply burdened | 3. Financially burdened | 4. Housing cost-burdened | 5. Financial & housing cost-burdened |
| --- | --- | --- | --- | --- | --- | --- |
|  | 1. Least materially burdened | - | 0.32***  (0.2 - 0.43) | 0.18***  (0.08 - 0.28) | 0.01  (-0.08 - 0.1) | 0.08***  (-0.05 - 0.22) |
|  | 2. Multiply burdened | -0.32***  (-0.43 - -0.2) | - | -0.14*  (-0.27 - 0.01) | -0.31***  (-0.43 - -0.18) | -0.23**  (-0.39 - -0.08) |
|  | 3. Financially burdened | -0.18***  (-0.28 - -0.08) | 0.14*  (-0.01 - 0.27) | - | -0.17**  (-0.28 - 0.06) | -0.1  (-0.24 - 0.05) |
|  | 4. Housing cost-burdened | -0.01  (-0.1 - 0.08) | 0.31***  (0.18 - 0.43) | 0.17***  (0.06 - 0.28) | - | 0.07  (-0.07 - 0.22) |
|  | 5. Financial & housing cost-burdened | -0.08  (-0.22 - -0.05) | 0.23**  (0.08 - 0.39) | 0.1  (-0.05 - 0.24) | -0.07*  (-0.22 - 0.07) | - |

*** p ≤0.001; ** p ≤0.01; * p ≤ 0.05
