# Supplementary material for: The association between material hardship and physical and mental health among older adults: Multi-channel sequence Approach
Source: PLoS One. 2025 Mar 10;20(3):e0319270. doi: 10.1371/journal.pone.0319270 (PMC11893115; doi:10.1371/journal.pone.0319270)
Supplement: Attachment — Submitted filename: PONE-D-24-42119_Tien Van Nguyen_Review.docx [file pone.0319270.s003.docx]

**General comments.**

Paper ID: **PONE-D-24-42119**

Paper Title: **The Association Between Material Hardship and Physical and Mental Health among Older Adults: Multi-Channel Sequence Approach**

Reviewer Name: Tien Van Nguyen

Date: 14 October 2024

**Review**

***First impressions***

Overall, the research design is sound and the writing is well-structured. The background section is excessively lengthy and requires condensation. The study provides interesting information about material hardship and its association with the well-being experienced by older adults. The paper was well structured with the appropriate language used. The study results addressed the research question and objective.

However, there are some specific issues within each section as detailed in the comments below.

***Introduction:*** Material hardship among older adults

The introduction was well-structured and provides comprehensive information regarding the paper, as well as was well organized and explained the importance of the study. Some points should be considered to improve the introduction:

Line 2 of Page 3: Authors should cite references.

Line 3 of Page 4: The author should add a few studies that have used multichannel chain analysis to simultaneously model complicated material trajectories, thereby demonstrating this method's usefulness.

***Methodology***

- Abbreviations: abbreviations noted the first time; From the second time onwards, only write that symbol: Health and Retirement Study (HRS); multi-channel sequence analysis (MCSA); ordinary least squares (OLS);....
- Statistical analyses:

+ Descriptive statistics: Applied to describe data about research subjects' information according to fall risk status: Number, rate (%); $\overline{X}$± SD for variables following normal distribution; Median (range) for variables that do not follow a normal distribution.

+ Inferential statistics: statistically significant with: *** p ≤0.001; ** p ≤0.01; * p ≤ 0.05 . Univariate and multivariate regression analysis to learn some factors related to the risk of falls in the elderly (statistically significant with 95%CI and *** p ≤0.001; ** p ≤0.01; * p ≤ 0.05).

***Results and discussion***

- In Table 1:

+ Education: Below high school (485) + High school (982) + Above High school (1160) ≠ 2628 participants

+ Work status (2018): Working (247) + Not working (2378) ≠ 2628 participants

- In Table 3, the authors should add a footnote of the table: chi-square and ANOVA test, *** p ≤0.001; ** p ≤0.01; * p ≤ 0.05.

***Conclusion***

Acceptable.

***References, tables and figures***

In the reference “Halpin, B. (2019)” shouldn’t delete DOI*.* Many references are outdated and need to be replaced by newer references.

Authors should conform to the format of the References as required by the Journal.

**Conclusion**

☒ Accept
