# Supplementary material for: The association between material hardship and physical and mental health among older adults: Multi-channel sequence Approach
Source: PLoS One. 2025 Mar 10;20(3):e0319270. doi: 10.1371/journal.pone.0319270 (PMC11893115; doi:10.1371/journal.pone.0319270)
Supplement: Attachment — Submitted filename: response letter.docx [file pone.0319270.s004.docx]

November 21, 2024

PONE-D-24-42119

The Association Between Material Hardship and Physical and Mental Health among Older Adults: Multi-Channel Sequence Approach

We greatly appreciate the opportunity to revise the above-referenced manuscript. We have reviewed the comments thoroughly and used them as a basis for our revisions. With these revisions, we hope that the manuscript may be considered for publication in PLOS ONE.

Please note that our responses are blue in standard format if they are presented in the letter only but italicized if they are included in the manuscript. In the revised manuscript, we highlighted in yellow any text that has been revised or added.

Reveiwer1

Please use the space provided to explain your answers to the questions above. You may also include additional comments for the author, including concerns about dual publication, research ethics, or publication ethics. (Please upload your review as an attachment if it exceeds 20,000 characters)

Reviewer #1: It’s an interesting and innovative study that identifies material hardship over time and examines its association with mental and physical health. Nevertheless, some minor points are unclear and, in some cases, need to be revised:
I recommend this manuscript for minor revision.

1. The relationship between material hardship and both mental and physical health can be considered in different contexts and over time. The current study uses available data from the HRS and LB modules; however, the outcomes and factors of interest may differ across time periods. Because material disadvantages accumulate and fluctuate over time, the authors should discuss and explain these cumulative consequences more clearly for the readers to understand.

We appreciate the constructive feedback. We agree with your perspective, and your point would be a great asset for future research that considers the cumulative consequences of accumulated material hardship. We have added this point as a limitation of this study as below.

*This study investigates the relationship between patterns of material hardship and later-life well-being, emphasizing the need for future research to examine cumulative effects for a more comprehensive understanding.*

2. How potential confounding factors are adjusted in the Ordinary Least Squares (OLS) regression analysis should be described more clearly

Thank you for this clarifying comment. We agree with your opinion and rewrite the confounding factors in the Ordinary Least Square (OLS) regression as below.

*Ordinary least squares (OLS) regression analysis was conducted to estimate the relationship between material hardship sequences and depressive symptoms or self-rated health. The OLS regression model included the independent variable (material hardship sequences), the dependent variables (depressive symptoms and self-rated health), and covariates (marital status, poverty status, gender, education, age, number of chronic diseases, race/ethnicity, and working status). All analyses were performed separately using Stata 14.0.*

Reviewer 2

General comments.

Paper ID: PONE-D-24-42119

Paper Title: The Association Between Material Hardship and Physical and Mental Health among Older Adults: Multi-Channel Sequence Approach

Reviewer Name: Tien Van Nguyen

Date: 14 October 2024

Review

*First impressions*

Overall, the research design is sound and the writing is well-structured. The background section is excessively lengthy and requires condensation. The study provides interesting information about material hardship and its association with the well-being experienced by older adults. The paper was well structured with the appropriate language used. The study results addressed the research question and objective.

However, there are some specific issues within each section as detailed in the comments below.

*Introduction:* Material hardship among older adults

The introduction was well-structured and provides comprehensive information regarding the paper, as well as was well organized and explained the importance of the study. Some points should be considered to improve the introduction:

Line 2 of Page 3: Authors should cite references.

Line 3 of Page 4: The author should add a few studies that have used multichannel chain analysis to simultaneously model complicated material trajectories, thereby demonstrating this method's usefulness.

We are grateful for this comment. We modified our manuscript as you recommended as below.

- Line 2 of Page 3: added citation
- Line 3 of Page 4: Added studies that have used multichannel analysis to highlight the importance of this approach as below.

*Multi-channel sequence analysis (MCSA) is a powerful methodological approach for examining complex, cumulative effects across various life domains. For example, Mattijssen and Pavlopoulos (2019) utilized MCSA to classify temporary employment careers in the Netherlands by integrating income and employment trajectories, unveiling diverse career patterns. Stafford et al. (2019) also applied MCSA to link work-family life-course patterns with later-life employment, highlighting the critical role of continuous employment and family timing.*

*Methodology*

- Abbreviations: abbreviations noted the first time; From the second time onwards, only write that symbol: Health and Retirement Study (HRS); multi-channel sequence analysis (MCSA); ordinary least squares (OLS);....
- Statistical analyses:

+ Descriptive statistics: Applied to describe data about research subjects' information according to fall risk status: Number, rate (%); $\overline{X}$± SD for variables following normal distribution; Median (range) for variables that do not follow a normal distribution.

+ Inferential statistics: statistically significant with: *** p ≤0.001; ** p ≤0.01; * p ≤ 0.05 . Univariate and multivariate regression analysis to learn some factors related to the risk of falls in the elderly (statistically significant with 95%CI and *** p ≤0.001; ** p ≤0.01; * p ≤ 0.05).

Thank you for your thoughtful feedback. We appreciate your perspective and have updated the abbreviations (HRS, MCSA, OLS) accordingly. Regarding your second comment, we have already presented the data using numbers, rates (%), mean ± SD, and inferential statistical significance denoted as ***p ≤ 0.001; **p ≤ 0.01; *p ≤ 0.05. Additionally, we would like to clarify that our research does not focus on fall risk.

*Results and discussion*

- In Table 1:

+ Education: Below high school (485) + High school (982) + Above High school (1160) ≠ 2628 participants

+ Work status (2018): Working (247) + Not working (2378) ≠ 2628 participants

- In Table 3, the authors should add a footnote of the table: chi-square and ANOVA test, *** p ≤0.001; ** p ≤0.01; * p ≤ 0.05.

Thank you for your insightful comment. Missing values were present in the education and work status variables and were addressed using multiple imputation, accounting for approximately 5% of cases in the final regression. Regarding Table 3, as it presents the results of a regression analysis, it is not appropriate to include chi-square or ANOVA tests. However, we have incorporated chi-square and ANOVA tests for Table 2 under the significance column, as per your suggestion.

*Conclusion*

Acceptable.

*References, tables and figures*

In the reference “Halpin, B. (2019)” shouldn’t delete DOI*.* Many references are outdated and need to be replaced by newer references.

Authors should conform to the format of the References as required by the Journal.

Conclusion

We sincerely appreciate your valuable feedback. The DOI for Halpin, B. (2019) has been retained, and the references have been updated to include more recent articles, except for the methodology references.
